# Cytogenetic analysis in three *Bryconamericus* species (Characiformes, Characidae): first description of the 5S rDNA-bearing chromosome pairs in the genus

**DOI:** 10.1186/1755-8166-6-13

**Published:** 2013-04-01

**Authors:** Diovani Piscor, Daniela Bocagini Ribacinko-Piscor, Carlos Alexandre Fernandes, Patricia Pasquali Parise-Maltempi

**Affiliations:** 1 Instituto de Biociências, Departamento de Biologia, Laboratório de Citogenética, Universidade Estadual Paulista “Júlio Mesquita Filho” (UNESP), Av. 24A, 1515, Rio Claro, SP, ZIP: 13506-900, Brazil; 2 Universidade Estadual de Mato Grosso do Sul (UEMS), BR 163, Km 20.2, Mundo Novo, MS, ZIP: 79980-000, Brazil

**Keywords:** Chromosomes, Heterochromatin, NOR, FISH, rDNA probe

## Abstract

**Background:**

Nowadays, the genus *Bryconamericus* is placed in subfamily Stevardiinae within of Characidae, but not shows consistent evidence of monophyletism. The purpose of this work was to study the chromosomes of three species of *Bryconamericus*, aiming to add cytogenetic knowledge and contribute to the understanding of the chromosomal evolution of this genus.

**Results:**

The chromosomes of three species of *Bryconamericus* were analyzed using cytogenetic techniques. The karyotype of *Bryconamericus stramineus* contained 6 metacentric (m) + 10 submetacentric (sm) + 16 subtelocentric (st) + 20 acrocentric (a), the fundamental number (FN) of 84, one silver impregnated (Ag-NOR) pair, one pair bearing the 18S ribosomal DNA sites, another pair bearing the 5S rDNA sites, and a few positive C-bands. *Bryconamericus turiuba* had a karyotype containing 8 m + 10sm + 14st + 20a (FN = 84), one chromosome pair Ag-NOR, two pairs bearing the 18S rDNA sites, two pairs bearing the 5S rDNA sites, and a few C-band regions. *Bryconamericus* cf. *iheringii* had a karyotype containing 10 m + 14sm + 18st + 10a (FN = 94), including one pair with a secondary constriction Ag-NOR positive. In this karyotype the fluorescent in situ hybridization (FISH) showed the 18S and 5S rDNA probe in adjacent position.

**Conclusions:**

The results obtained in this work showed different characteristics in the organization of two multigene families, indicating that distinct evolutionary forces acting on the diversity of rDNA sequences in the genome of three *Bryconamericus* species.

## Background

*Bryconamericus* is a genus of the family Characidae that is widely distributed across Central America and South America. According to the published data there are about 70 species described in the genus *Bryconamericus*[1]. New species have been described, including a species from French Guyana known as *Bryconamericus guyanensis* sp. n. [2], so the number of known species is certainly higher than previously estimate.

The phylogeny of the genus *Bryconamericus* has been discussed by several authors. According to Géry [3] the genus *Bryconamericus* belonged to the subfamily Tetragonopterinae. Later, the *Bryconamericus* species were included in Characidae *incertae sedis* by Lima et al. [4]. More recently Mirande [5] and Oliveira et al. [6] showed that *Bryconamericus* can be a polyphyletic group, but included within of the subfamily Stevardiinae (Family Characidae) and no more in *incertae sedis* as proposed by Lima et al. [4].

Cytogenetic data for the genus *Bryconamericus* have been described in the literature by some authors, i.e., chromosomal analyses by Giemsa, detection of major ribosomal DNA (45S), and differential staining, such as C-bands, G-bands and chromomycin A_3_ (CMA_3_) [7-10]. In these studies, the most frequently reported diploid number was 2n = 52 chromosomes; however, variation involving the chromosome morphology and the number and location of NORs were also registered. For example, Capistano et al. [11] identified three distinct cytotypes and a significant variability of NOR in three populations of *Bryconamericus* aff. *iheringii*, as follows: the cytotype I (Maringá Stream) was 12 m + 18sm + 8st + 14a, with two to four NOR-positive chromosomes and six chromosomes carrying 18S rDNA sites; the cytotype II (Keller River) was 8 m + 28sm + 6st + 10a, with two to four NOR-positive chromosomes and ten chromosomes carrying 18S rDNA sites; the cytotype III (Tatubepa Stream) was 8 m + 20sm + 8st + 16a, with two NOR-positive chromosomes and two chromosomes carrying 18S rDNA sites.

Portela-Castro et al. [9] analyzed a population of *B.* aff. *iheringii* from Keller River in the upper Paraná River basin (State of Paraná, Brazil) using conventional Giemsa staining and several banding techniques (C, G and R), which identified two cytotypes, i.e., the cytotype I with 12 m + 18sm + 8st + 14a and the cytotype II with 8 m + 28sm + 6st + 10a. Two cytotypes were also reported in a population of *Bryconamericus* aff. *exodon* from Três Bocas Stream (Tibagi River basin, state of Paraná, Brazil), i.e., cytotype I - 16 m + 12sm + 6st + 18a with a FN = 86, cytotype II - 10 m + 24sm + 6st + 12a and FN = 92 [12].

The aim of this study was to accomplish cytogenetic analyses and show for first time the location of 5S rDNA sequences in three species of the genus *Bryconamericus*, in order to obtain a better knowledge about the chromosomal characteristics and contribute to understanding of the chromosomal evolution of this genus.

## Results

All individuals of the all three studied species showed the diploid number of 2n = 52 chromosomes but a different karyotypic formulae among the species. There were no karyotypic differences between the sexes.

**Figure 1:**
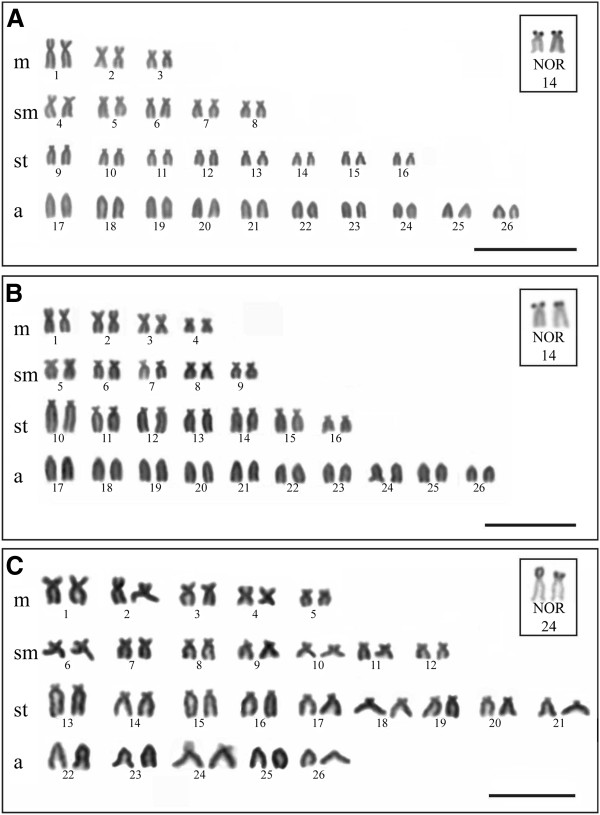
**Giemsa stained chromosomes. A.***B. stramineus*. **B.***B. turiuba*. **C.***B.* cf. *iheringii*. The boxes show the chromosomal pairs carriers of Ag-NORs of each species. Bar = 10 μm.

**Figure 2:**
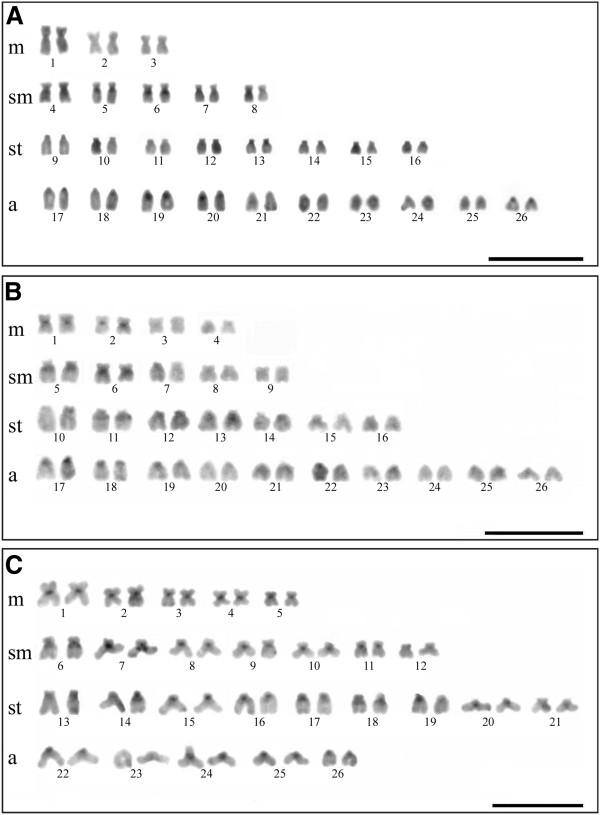
**C-banded chromosomes. A.***B. stramineus*. **B.***B. turiuba*. **C.***B.* cf. *iheringii*. Bar = 10 μm.

**Figure 3:**
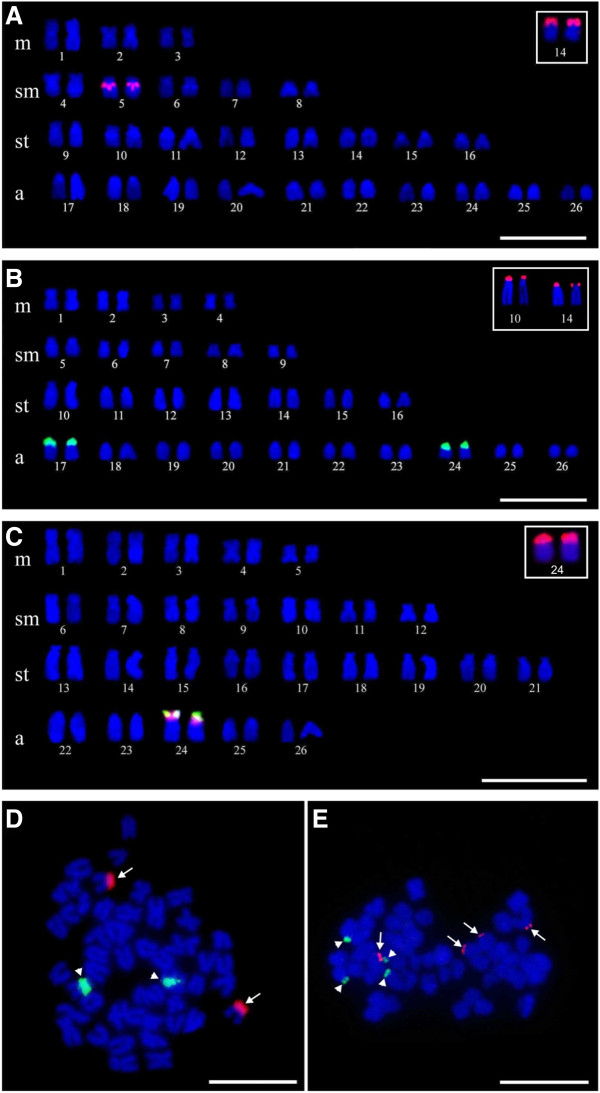
**Fluorescent in situ hybridization (FISH). A.** FISH with 5S rDNA probe in *B. stramineus*. **B.** FISH with 5S rDNA probe in *B. turiuba*. **C.** Double-FISH with 5S rDNA probe (green) and 18S (red) in *B.* cf. *iheringii*. The boxes show the 18S rDNA signals. **D.** Double-FISH in *B. stramineus*. **E.** Double-FISH in *B. turiuba*. The arrows indicate the signals of 18S rDNA and the arrow heads indicate the signals of 5S rDNA. Bar = 10 μm.

*Bryconamericus stramineus* specimens had a karyotype of 6 m + 10sm + 16st + 20a (FN = 84) and the homologous chromosome of pair 14 was Ag-NOR positive (Figure 1A). Positive C-band heterochromatin was detected in the centromeric and pericentromeric regions of three and four pairs, respectively (Figure 2A). FISH using rDNA probes detected 18S rDNA sites in the terminal position of the short arm of the subtelocentric pair 14, and 5S rDNA sites in the pericentromeric region of the submetacentric pair 5 (Figure 3A). The double-FISH technique was used to confirm that the two rDNA (18S and 5S) clusters were physically located in different chromosome pairs (Figure 3D).

All *Bryconamericus turiuba* specimens had a karyotype with 8 m + 10sm + 14st + 20a (FN = 84) and the silver ion impregnated in the terminal region of the short arm of the subtelocentric pair 14 (Figure 1B). Heterochromatin detected by the C-band technique was evident in the centromeric and pericentromeric regions of five and four pairs, respectively (Figure 2B). The FISH technique identified the 18S rDNA in two pairs: one in the short arm of pair 14, which coincided with the Ag-NOR pair, and another in the terminal region of the pair 10 (Figure 3B). 5S rDNA sites were detected in the pericentromeric region of the acrocentric pairs 17 and 24 (Figure 3B). The double-FISH technique showed that these two rDNA clusters were not syntenic in *B. turiuba* (Figure 3E).

**Figure 4:**
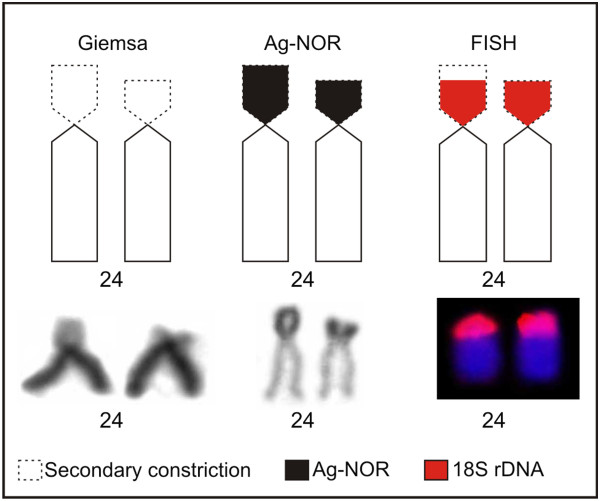
**Pair 24 of *B. *cf. *iheringii *submitted to the Giemsa staining, silver impregnation and FISH with 18S rDNA probe.** Observe the size heteromorphism of the secondary constriction and Ag-NOR.

The karyotype of *Bryconamericus* cf. *iheringii* specimens was 10 m + 14sm + 18st + 10a (FN = 94); the acrocentric pair 24 exhibited a size heteromorphism regarding to secondary constriction and Ag-NOR clusters in the metaphase cells of all sampled specimens (Figure 1C). After C-band technique, heterochromatin was visualized in the centromeric and pericentromeric regions of 21 and four pairs, respectively (Figure 2C). FISH using the 18S rDNA probe detected a site in the terminal region of the short arm of the pair 24 (Figure 3C). The 5S rDNA sites were detected in the short arm of pair 24 and the double-FISH showed that the 45S and 5S rDNA clusters were adjacent (Figure 3C). The Ag-NOR-size heteromorphism observed in impregnated silver chromosomes was not detected when the chromosomes were submitted to the FISH technique using 18S rDNA probe (Figure 4).

**Figure 5:**
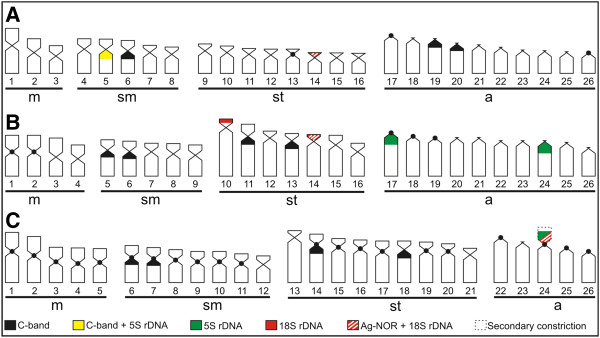
**Ideograms of three *Bryconamericus *species, showing the constitutive heterochromatin, Ag-NOR, 18S and 5S rDNA distribution pattern. A.***B. stramineus*. **B.***B.turiuba*. **C.***B.* cf. *iheringii*.

The ideogram summarizes all markers on chromosomes of the three *Bryconamericus* species in this study (Figure 5).

## Discussion

The karyotypes of *B. stramineus* and *B. turiuba* were similar in terms of their acrocentric chromosome number and they shared the same FN (84). One karyotype very similar was described for *B. stramineus* from Mogi-Guaçu River (State of São Paulo, Brazil) with 26 m/sm and 26st/a chromosomes [13]. By contrast, *B.* cf. *iheringii* had half the acrocentric chromosome number and the FN was higher (94) compared with the other two species. Other populations of *B.* aff. *iheringii* from rivers belonging to state of Paraná (Brazil) showed fundamental numbers 88, 90, 92 and 94 [8,9,11].

Further relevant features were also detected when the positive C-band heterochromatin regions were compared, including blocks in the pericentromeric region of some submetacentric pairs, which seem to be conserved among the three studied species; for example, heterochromatin blocks in pairs 5 and 6 in *B. stramineus*, 5 and 6 in *B. turiuba,* and 6 and 7 in *B.* cf. *iheringii*, as well as in pairs 11 and 13 in *B. turiuba* and 14 and 18 in *B.* cf. *iheringii*. Thus, the similar position of these blocks indicates that this heterochromatin could be conserved and include similar repetitive DNA in the genome these three species.

Different Ag-NOR pairs were also detected, which differed in size in *B. stramineus* and *B. turiuba*, and in morphology in *B.* cf. *iheringii*. NORs were detected using both silver impregnation and 18S rDNA probes. Only one pair carrier of NOR was detected in *B. stramineus* and *B.* cf. *iheringii* with both techniques, whereas in *B. turiuba* two pairs were detected by FISH (18S rDNA probe) indicating that in all cells the Ag-NORs in the pair 10 were inactive. Ag-NORs identified by silver impregnation only correspond to sites that were active in previous interphase, which explains the additional pair (pair 10) observed in *B. turiuba* when the FISH technique was employed.

The Ag-NOR size heteromorphism found in pair 24 of the *B.* cf. *iheringii* karyotype coincided with the secondary constriction (Figure 4). This may indicate a difference in 45S rDNA gene transcription, because the 18S rDNA FISH probe of these regions detected signals with the similar size. As discussed by Arruda and Morielle-Versute [14], the size heteromorphism of the secondary constriction and Ag-NOR located in the chromosome of pair 8 of the amphibian *Leptodactylus podicipinus*, can be mainly attributed to the difference in the degree of condensation between homologues chromosomes and differential gene activity of the rDNA segments (45S), taking into account that and such no heteromorphism was observed in the signal strength of fluorescence in situ hybridization with rDNA probes. This could explain the difference between the size of the Ag-NOR and the FISH (18S rDNA probe) found in the pair 24 of *B.* cf. *iheringii* in this study. Differently, other population of *B.* aff. *iheringii* (Cytotype III) from Tatupeba Stream (State of Paraná, Brazil) showed one chromosome pair with NOR-size heteromorphism after the FISH technique using 18S rDNA probe [11].

The chromosomal location of the 5S rDNA sequences is described for the first time in *Bryconamericus* and also provided a useful cytological marker to comparisons among the three species. From an evolutionary viewpoint, these data are intriguing because the insertion of transposable elements in 5S rDNA sequences could lead to the dispersion of these sequences in different chromosomes via transposition in *B. stramineus*, *B. turiuba,* and *B.* cf. *iheringii*. According Raskina et al. [15], transposable elements have been proposed as one of the mechanisms responsible for the process of mobility of rDNA sequences to new sites.

The 45S gene family is transcribed in the nucleolus, whereas the 5S gene family is transcribed outside the nucleolus, suggesting that functional differences may require different physical locations of these two multigenic families [16]. Gene conversion and unequal crossing-over could be important processes in the maintenance of a conserved sequence in multiple tandem arrays [17]. These mechanisms might be more efficient regardless of whether 45S and 5S clusters remain separated from each other instead of in a linked configuration, avoiding disruptive interferences, such as undesired translocation of 5S sequences inside the 45S arrays [18], and explaining why this sequences are in different chromosomes in *B. stramineus* and *B. turiuba*, such as most vertebrates, and not in synteny.

Unlike the other two species analyzed in this study, the chromosomes of *B.* cf. *iheringii* showed a physical position adjacent of the 18S and 5S rDNA genes in the pair 24. This characteristic synteny was also reported in a population of *Astyanax scabripinnis*, where one of the chromosome pairs that carried the 5S rDNA also carried NORs [19]. According to Schweizer and Loidl [20], telomeric regions are propitious to genetic material transference due to their proximity within interphasic nucleus, promoted by the ordering of chromosomes based on the Rabl’s model. Hence, in the case of *B.* cf. *iheringii*, the 45S rDNA transfer events near 5S rDNA or vice versa could be facilitated.

Diniz et al. [21] showed that *Triportheus nematurus* (Characidae) had three pairs of NORs, two of which were co-located adjacent to the 5S rDNA. Species of the family Salmonidae, such as *Salmo salar* and *Oncorhynchus mykiss,* had syntenic 45S and 5S sites, suggesting that were likely to have co-evolved [22,23]. The authors argued that this synteny could be attributable to a translocation of 18S rDNA sequences within the 5S rDNA or vice versa in *T. nematurus*, while chromosomal rearrangements in *S. salar* and *O. mykiss* may have contributed to this feature after the divergence of the two species.

## Conclusions

The cytogenetic data identified in this work indicate that the three fish species showed conserved and divergent characteristics which could facilitate an understanding of the evolutionary dynamic of each genome. However, the organization of the rDNA observed here, can be indicate that there are distinct evolutionary forces acting in the diversification of these sequences in the genome of the three *Bryconamericus* species.

## Materials and methods

### Sampling

In this study, seven specimens of *Bryconamericus turiuba* Langeani et al. [24] (five males and two females) and five *Bryconamericus* cf. *iheringii* Boulenger, 1887 (all males) were collected from a tributary of the Passa-Cinco River (22°23'25.4''S, 47°50'47.8''W) and a tributary of the Corumbataí River (22°16'6.2''S, 0.47°37'16.9''W), respectively, in the Corumbataí River basin in the state of São Paulo, Brazil. Specimens of *Bryconamericus stramineus* Eigenmann, 1908 (12 males and nine females) were collected from Guaçu Stream (23°54'19.6''S, 54°21'43.4''W) in the Iguatemi River basin, state of Mato Grosso do Sul, Brazil.

### Cytogenetics

Chromosomes were obtained from cells in the anterior and posterior regions of the kidney, according to the methodology proposed by Foresti et al. [25]. The NORs were detected using the silver ion impregnation technique described by Howell and Black [26], while heterochromatin was observed using the C-band technique proposed by Sumner [27]. Based on the most common classification system used for fish chromosomes, the morphology of chromosomes was determined according to the arms, where the length of the long arm (q) was divided by the length of the short arm (p). Chromosomes with two arms were classified as metacentric (m) with arm rate between 1–1.7, submetacentric (sm) with arm rate between 1.71-3 and subtelocentric (st) with arm rate between 3.01-7, whereas chromosomes with a single arm were considered acrocentric (a) with arm rate above 7.

### DNA extraction and production of probes

Genomic DNA was extracted from fin samples of *Bryconamericus* according to Sambrook and Russell [28]. The 18S rDNA probe was obtained by PCR (Polymerase Chain Reaction) using the primers (NS1 = 5^′^-GTAGTCATATGCTTGTCTC-3^′^ and NS8 = 5^′^-TCCGCAGGTTCACCTACGGA-3^′^) described by White et al. [29] while the 5S rDNA probe was obtained by PCR using the primers (A = 5^′^-TACGCCCGATCTCGTCCGATC-3^′^ and B = 5^′^-CAGGCTGGTATGGCCGTAAGC-3^′^) described by Pendás et al. [22] and Martins and Galetti Jr. [18].

### Fluorescent in situ hybridization (FISH)

The FISH technique used 18S and 5S rDNA probes tagged with digoxigenin-11-dUTP (Roche) and biotin-16-dUTP (Roche), according to the method of Pinkel et al. [30] with modifications described in Marreta et al. [31]. The analysis was performed in the Cytogenetic Laboratory in Universidade Estadual Paulista “Júlio de Mesquita Filho” (UNESP), Rio Claro, São Paulo, Brazil. Slides prepared with metaphase chromosomes were incubated with RNase (20 ng/μL) for 1 h at 37°C and dehydrated using an alcohol series (70%, 90%, and 100%). Chromosomal DNA was denatured for 4 min in 70% formamide in 2× standard sodium citrate (SSC) at 70°C and dehydrated immediately using an alcohol series (70%, 90%, and 100%). The hybridization solution (20 μL of 50% formamide in 2× SSC, 10% dextran sulfate, and 6 ng/μL rDNA probe) was incubated for 10 min at 95°C and applied to each slide. After overnight hybridization at 37°C, the slides were washed twice for 5 min with 50% formamide in 2× SSC (pH 7.0) at 45°C, twice with 1× SSC for 5 min at 45°C, and once with 4× triton (1 part 20× SSC, 4 parts H_2_O and 250 mL 100× triton) for 5 min. Signals were detected using antidigoxigenin-rhodamine (Roche) for digoxigenin and avidin-FITC (Sigma) for biotin. The chromosomes were counter-stained using Vectashield Mounting Medium and DAPI (4’,6-diamidino-2-phenylindole), before visualization with an Olympus BX51 microscope coupled to a digital camera (Olympus model D71). Images were captured using the DP Controller program.

## Abbreviations

Ag-NOR: Silver nitrate staining; NOR: Nucleolar organizer region; FN: Fundamental number; CMA3: Chromomycin A_3_; DAPI: 4',6-diamidino-2-phenylindole; 2n: Diploid number; m: Metacentric chromosome; sm: Submetacentric chromosome; st: Subtelocentric chromosome; a: Acrocentric chromosome; p: Short arm; q: Long arm; FISH: Fluorescence in situ hybridization; rDNA: Ribosomal DNA; rRNA: Ribosomal RNA; PCR: Polymerase chain reaction

## Competing interests

The authors declare that they have no competing interests.

## Authors’ contributions

DP collected the animals, performed the cytogenetic studies and drafted the manuscript. DBRP performed the DNA extraction and production of probes. CAF performed some cytogenetic preparations. PPPM supervised the experiments studies, helped draft the manuscript and revised the final text. All authors read and approved the final manuscript.
